# Automated Annotation of Untargeted All-Ion Fragmentation LC–MS Metabolomics Data with MetaboAnnotatoR

**DOI:** 10.1021/acs.analchem.1c03032

**Published:** 2022-02-18

**Authors:** Gonçalo Graça, Yuheng Cai, Chung-Ho E. Lau, Panagiotis A. Vorkas, Matthew R. Lewis, Elizabeth J. Want, David Herrington, Timothy M. D. Ebbels

**Affiliations:** † Section of Bioinformatics, Division of Systems Medicine, Department of Metabolism, Digestion and Reproduction, Imperial College London, South Kensington Campus, Sir Alexander Fleming Building, London SW7 2AZ, U.K.; ‡ Department of Epidemiology and Biostatistics, School of Public Health, Imperial College London, London W2 1PG, U.K.; § Section of Biomolecular Medicine, Division of Systems Medicine, Department of Metabolism, Digestion and Reproduction, Imperial College London, South Kensington Campus, Sir Alexander Fleming Building, London SW7 2AZ, U.K.; ∥ Institute of Applied Biosciences, Centre for Research and Technology Hellas, Thessaloniki 57001, Greece; ⊥ Section of Bioanalytical Chemistry and National Phenome Centre, Division of Systems Medicine, Department of Metabolism, Digestion and Reproduction, Imperial College London, Hammersmith Campus, IRDB Building, London W12 0NN, U.K.; # Section on Cardiovascular Medicine, Wake Forest School of Medicine, Winston-Salem, North Carolina 27157, United States

## Abstract

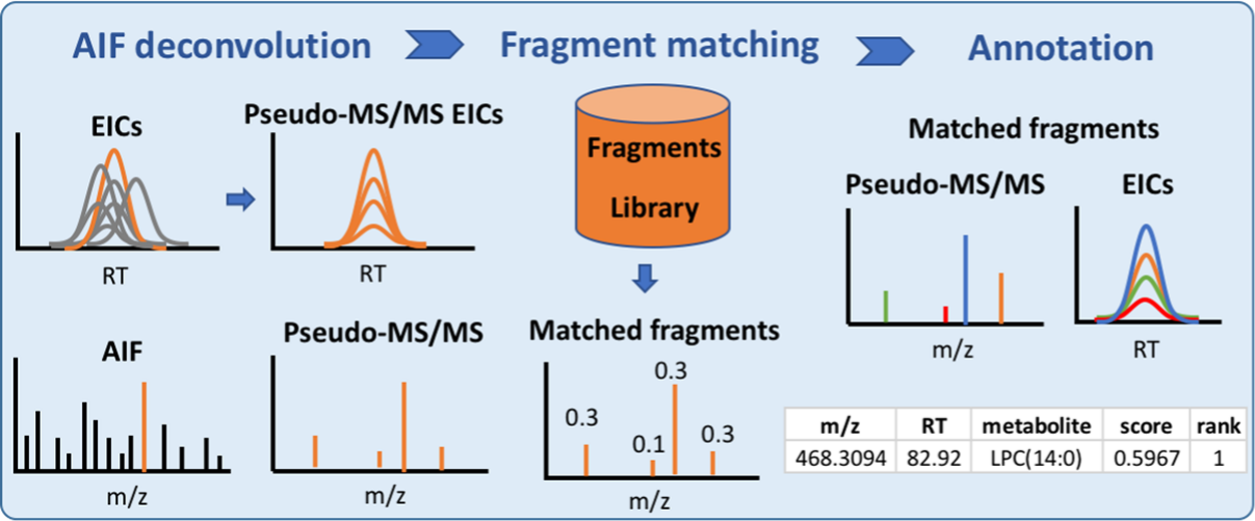

Untargeted metabolomics and lipidomics LC–MS experiments produce complex datasets, usually containing tens of thousands of features from thousands of metabolites whose annotation requires additional MS/MS experiments and expert knowledge. All-ion fragmentation (AIF) LC–MS/MS acquisition provides fragmentation data at no additional experimental time cost. However, analysis of such datasets requires reconstruction of parent–fragment relationships and annotation of the resulting pseudo-MS/MS spectra. Here, we propose a novel approach for automated annotation of isotopologues, adducts, and in-source fragments from AIF LC–MS datasets by combining correlation-based parent–fragment linking with molecular fragment matching. Our workflow focuses on a subset of features rather than trying to annotate the full dataset, saving time and simplifying the process. We demonstrate the workflow in three human serum datasets containing 599 features manually annotated by experts. Precision and recall values of 82–92% and 82–85%, respectively, were obtained for features found in the highest-rank scores (1–5). These results equal or outperform those obtained using MS-DIAL software, the current state of the art for AIF data annotation. Further validation for other biological matrices and different instrument types showed variable precision (60–89%) and recall (10–88%) particularly for datasets dominated by nonlipid metabolites. The workflow is freely available as an open-source R package, MetaboAnnotatoR, together with the fragment libraries from Github (https://github.com/gggraca/MetaboAnnotatoR).

## Introduction

Liquid chromatography–mass spectrometry (LC–MS)-based untargeted metabolomics and lipidomics experiments are widely used approaches for biomarker discovery and to study disease mechanisms. They typically result in complex datasets containing thousands to tens of thousands of features [mass-to-charge ratio–retention time (*m*/*z*–RT) pairs], corresponding to adducts, in-source fragments, multimers, and isotopologues.^1^ The numbers of compounds corresponding to such features are of the order of thousands.^1,2^ Biological interpretation of the data depends wholly on annotation of each feature to a chemical structure. Despite its utmost importance to the field, this annotation step is a key bottleneck in untargeted metabolomics data analysis and interpretation.

Accurate metabolite annotation is a largely manual and time-consuming process that typically consists of MS spectral inspection and additional MS/MS-targeted fragmentation experiments on the features of interest, usually run post acquisition. Data-independent acquisition (DIA) LC–MS schemes, such as alternating low and high collision energy (parallel) acquisition without prior ion selection (referred to henceforth as all-ion fragmentation, AIF, and also known as MS^E^, MS/MS^ALL^, or bbCID), sequential window acquisition of all theoretical mass spectra (SWATH-MS),^3^ and rapidly scanning quadrupole (SONAR)^4^ have been developed to provide analysts with fragmentation data acquired during regular LC–MS runs. These reduce the need for additional MS/MS fragmentation experiments.^1,5^ In some cases, structural information can also be accessed from the MS spectrum of some compounds in the form of in-source fragments, which are formed during ionization.^5,6^ Nevertheless, to get meaningful information from AIF datasets, special processing routines are needed to reconstruct the parent–fragment ion relationships and obtain a spectrum containing all fragments arising from the same parent (pseudo-MS/MS spectrum). Several commercial and open-source/free software packages, such as MS-DIAL,^7^ DIA-Umpire,^8^ RAMClustR,^5^ and R-MetaboList,^9^ employ deconvolution techniques to link AIF parent ions to their corresponding fragment ions and extract the corresponding pseudo-MS/MS spectra. Two R packages, CAMERA^10^ and RAMClustR,^5^ can also be used to reconstruct in-source parent-fragment pseudo-MS/MS spectra. However, the interpretation of the resulting pseudo-MS/MS spectra still requires expert knowledge and manual verification to determine compound identity from the underlying features.

To identify features belonging to known compounds, researchers have built metabolite spectral libraries containing thousands of MS/MS spectra and developed spectral matching algorithms to annotate unknowns using spectral similarity. Notable examples include the databases METLIN,^11^ MassBank,^12^ and GNPS^13^ which are invaluable in the identification of metabolites from diverse sample matrices. Additional approaches to MS/MS interpretation involve spectral matching to *in silico* spectra generated from metabolite structures, therefore covering a larger chemical space than spectral databases.^14−18^ Most of these tools are, however, most suited to single-ion MS/MS, rather than AIF MS/MS data.

In most automated AIF annotation software tools, such as LipidMatch,^19^ R-MetaboList,^9,20^ or MS-DIAL,^7^ the parent–fragment ion relationship is determined by inspecting which fragment peaks co-elute with the parent ions. This is usually conducted using deconvolution strategies consisting of matching RTs at the maximum intensity and height at half-maximum (peak shape matching) or by calculating the correlation of peak intensity shapes across a fixed RT window (peak shape correlation). These can result in incomplete pseudo-MS/MS spectra, particularly for co-eluting compounds containing similar types of chemical groups, which upon fragmentation produce nonspecific fragment ions (e.g., lipid head groups or glucuronides in conjugated metabolites) or for poorly detected fragment peaks. In these cases, peak shape correlations are affected because the shapes of parent and fragment peaks are different.

Nonetheless, peak shape matching and peak shape correlation approaches when used together with fragment library matching could improve the annotation of AIF LC–MS features. Most open-source tools for AIF LC–MS annotation attempt to annotate all detected features. This can be time-consuming and could miss low-intensity features which might be relevant for the question under study. In addition, isotopologues, in-source fragments, and some adducts are not always considered. Since in most untargeted studies, statistical analysis is run prior to feature annotation, focusing on a subset of features (e.g., those which reach statistical significance) might save time and simplify the process. In addition, several strategies have been developed specifically to annotate lipid features using AIF data, such as Lipid-Pro,^21^ Arcadiate,^22^ and LipidMatch,^19^ which can annotate peak lists but only for lipid and lipid-like features. R-MetaboList^9,20^ focuses mostly on small molecules, while MS-DIAL^7^ allows the analysis not only of both lipids and smaller metabolite molecules but only of monoisotopic features.

Here, we propose a new workflow for the annotation of lipidomics and metabolomics LC–MS AIF datasets based on pseudo-MS/MS reconstruction and matching of the fragment ions to fragment databases. The workflow is designed to annotate features obtained from LC–MS experiments processed using software such as XCMS^23^ or MZmine,^24^ which may include all types of ions, namely, isotopologues, adducts, and in-source fragments. The workflow has been implemented as an open-source R package MetaboAnnotatoR, providing tools for automated annotation, reporting, and visualization. We also provide an extensive fragment library for lipids (based on theoretical *m*/*z* values of the expected fragments adapted from LipidMatch R package^19^) and other small molecules (based on experimental MS/MS spectra) which can be expanded and tailored by the user to their own requirements. The software and libraries are available from Github (https://github.com/gggraca/MetaboAnnotatoR).

## Experimental Section

### Implementation of the Automated Annotation Workflow

**Figure 1:**
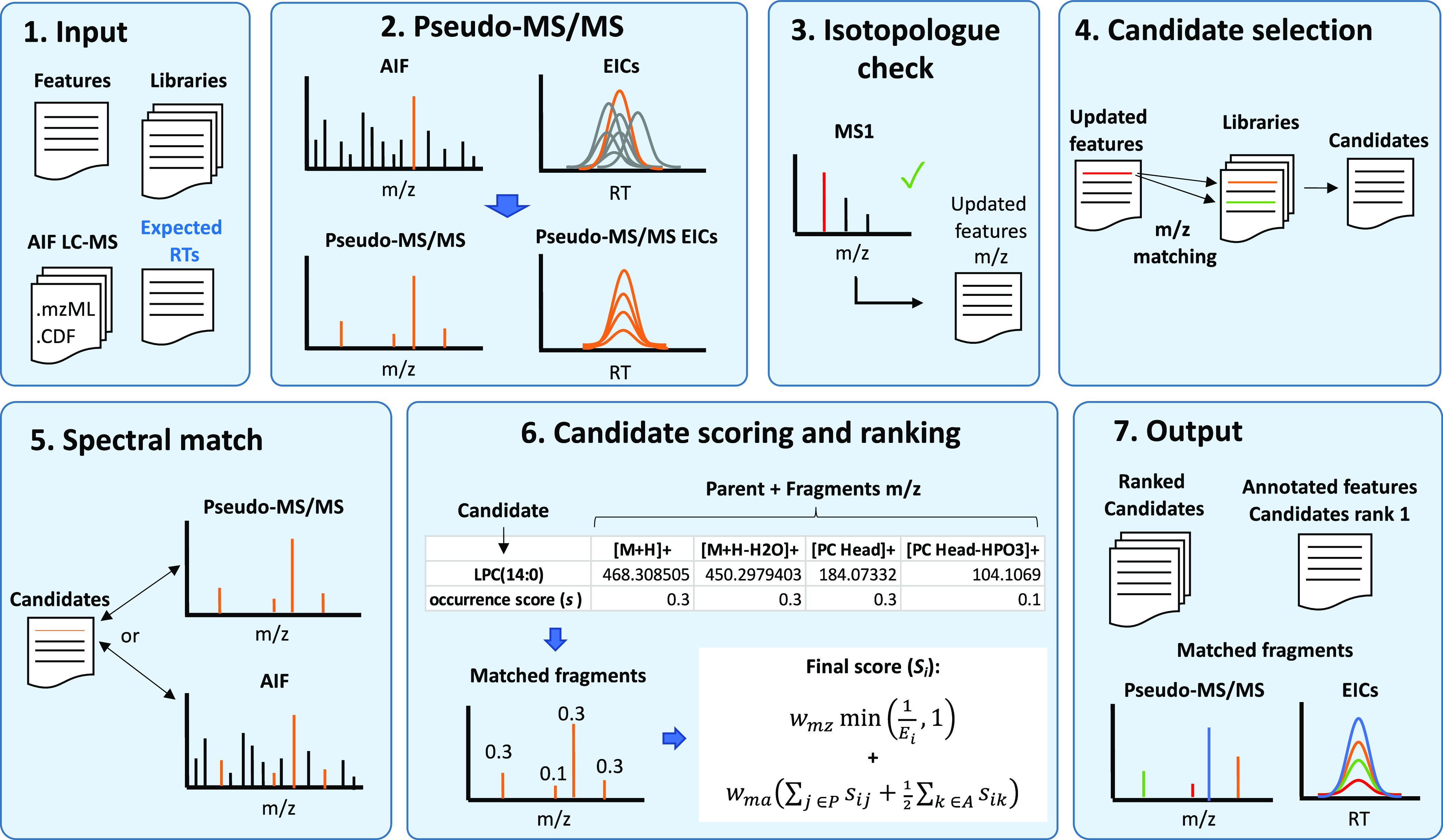
Schematic of MetaboannotatoR workflow for AIF LC–MS feature annotation.

The workflow was designed for feature-by-feature annotation on one or more LC–MS AIF samples, as illustrated in Figure 1.

The workflow can be divided into seven steps detailed below:

(1) Data input. The workflow starts with a table of features (*m*/*z* and RT values) to be annotated (target features) and samples where the target features have been detected. This is used to load the raw AIF LC–MS data which should be in the vendor-neutral formats mzML or netCDF. Alternatively, a RAMClustR^5^ R object containing deconvolved pseudo-MS/MS spectra for the features can also be used as data input. Positive and negative ionization modes for the same chromatography are analyzed separately, to make effective use of mode-specific reference libraries. The files containing the reference libraries of candidate parent and fragment *m*/*z* values are also loaded. Optionally, expected RT windows for particular metabolite classes can be loaded to further restrict the number of candidate matches.
(2) Pseudo-MS/MS generation. For each target feature, we obtain the extracted ion chromatogram (EIC) from the no-fragmentation (MS1) scans and all EICs from the AIF scans in the same RT window. We then calculate the Pearson correlation coefficient r, between the intensity of the feature EIC (MS1) and each EIC from the AIF ions. The peaks from the AIF spectra with correlation greater than a threshold θ defined by the user (usually 0.7–0.8, default 0.8) are gathered to form the pseudo-MS/MS spectrum. If a RAMClustR object is used, the target feature *m*/*z* and RT values will be used to locate the corresponding pseudo-MS/MS spectrum (cluster).^5^ If no pseudo-MS/MS spectrum is obtained (i.e., no peaks with *r* > θ), the full AIF spectrum at the feature RT is used as the pseudo-MS/MS spectrum.
(3) Isotopologue check. The workflow was designed to annotate features including isotopologue peaks, adducts, and in-source fragments. Therefore, the type of isotopologue must be determined to ensure that only the monoisotopic mass of each isotopic distribution is used in the search for candidates. This is important because the fragment database is composed solely of monoisotopic masses. Currently, the workflow only considers carbon isotopologues. In an isotopic distribution of the same compound, the peak intensities will be highly correlated, and for small molecules, the monoisotopic peak is expected to have the highest intensity. The peaks of MS1 spectra within the same RT window as the target feature that show a high EIC correlation (e.g., *r* > 0.8) with the target feature are inspected. The intensity of the target feature is then compared with that of the peak at target *m*/*z*—1 Da if one exists. If the target feature intensity is higher, then it is assumed to represent the monoisotopic mass. Otherwise, the feature intensity will be iteratively compared with the intensity of the peak at target feature *m*/*z*—2 Da (then −3 Da and so on) until the peak with the highest intensity (monoisotopic peak) is found. The *m*/*z* difference between the feature *m*/*z* and the monoisotopic peak will define the type of isotopologue. Typically, most of the LC–MS peaks obtained from XCMS outputs are either monoisotopic (M + 0) or correspond to the second (M + 1), third (M + 2), and fourth (M + 3) peaks of the isotopic distribution.
(4) Candidate selection. The monoisotopic mass of the target feature is used to search for candidate metabolite(s) in the library. The libraries are organized as a collection of .csv files, one for each combination of a metabolite or class of metabolites (lipids) and an adducting species (e.g., Na^+^ and K^+^). Each entry in a library file contains the (parent) metabolite name and adduct *m*/*z* and expected fragment *m*/*z* values (Figure 1, step 6). The corrected target monoisotopic *m*/*z* (from step 3) is used for a first screening of candidates. If no match is found, a second search is performed through the library fragment *m*/*z* values in case the target feature is an in-source fragment. In both cases, a tolerance of 25 ppm is used (default for Q-ToF instruments, users can modify this).
(5) Spectral match. For each candidate, the *m*/*z* of adduct and fragments will be compared with those of pseudo-MS/MS peaks using a 0.01 Da tolerance (default for Q-ToF type instruments, modifiable by the user). If some of the expected fragments from a candidate are not matched to the pseudo-MS/MS peaks or if no pseudo-MS/MS spectrum was generated, the AIF spectrum will be searched for these remaining fragments. This second search allows for fragments shared by co-eluting molecules which may not be captured by pseudo-MS/MS (e.g., head groups of some phospholipids) to be accounted for in the matching process.
(6) Candidate scoring and ranking. The score indicating the quality of the match between a library candidate and a target feature is composed of two parts: one measuring the similarity in *m*/*z* between the target and candidate and one gauging the similarity of the fragmentation patterns: *S* = *S*_mz_ + *S*_ma_. For the former, we use the reciprocal of the *m*/*z* error between the target and candidate parent, *E* (in ppm) bounded at unity: *S*_mz_ = min(1/*E*, 1). For the latter, each parent and fragment in the library is given an occurrence score (*s*, Figure 1). These scores reflect the likelihood of observing the fragment and allow the user to include metabolites in the library whose fragmentation can only be predicted in silico or obtained from the literature. The match score *S*_ma_ is then the sum of occurrence scores for all fragments which match pseudo-MS/MS. Where fragments are matched to the AIF, these are downweighted by a factor of two, reflecting their lower specificity compared to pseudo-MS/MS matches. Thus, for the *i*th candidate, the final score combines the two contributions
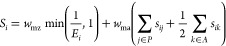
1where the weights *w*_mz_ = 1 – *w*_ma_ (default both 0.5) allow users to vary the influence of each subscore, balancing *m*/*z* errors with fragmentation pattern matching. Here, *P* denotes the set of all matches to the pseudo-MS/MS spectrum and A is the set of matches to the AIF spectrum.

(7) Output. The matches for each target feature are compiled and output to a table (.csv) and graphical outputs are saved as .pdf files. For each target feature, a list of the candidates ranked by score is produced, which includes the metabolite name, *m*/*z* matched, *m*/*z* error (ppm), the number of matched fragments, and score. Additionally, a list containing all rank 1 annotations is produced for quick result inspection. Graphical outputs contain, for each ranked result, the pseudo-MS/MS spectrum comprising the matched peaks and the corresponding EICs; and for each target feature, the pseudo-MS/MS and pseudo-MS (MS1 spectrum containing all ions correlated to the target feature) spectra and the corresponding EICs are stored for later inspection. If RAMClustR is used, no EICs are produced, as in this case, the annotation is performed using preprocessed pseudo-MS/MS data.

### Metabolite Fragment Libraries

We prepared libraries of metabolites, which consist of records that include parent ion *m*/*z* and expected MS/MS fragments from positive and negative electrospray ionisation (ESI) experiments. The libraries include both lipids and small-molecule (nonlipid) metabolites observed in mammalian biofluids and tissues. The lipid libraries were adapted from those of the LipidMatch R package,^19^ which is a library of theoretical *m*/*z* values for experimentally observed lipid fragments. The libraries were adapted to retain only fragments that were commonly observed experimentally in ESI MS/MS spectra and well-documented in the literature.^25,26^ Overall, 90,425 lipid entries, comprising several combinations of adducts, observed in both positive and negative modes of ionization were included in the library. These included adducts of H^+^, Na^+^, K^+^, and NH_4_^+^ for the positive mode and adducts of Cl^–^, HCOO^–^, CH_3_COO^–^, and H_2_PO_4_^–^ and the characteristic proton loss for the negative mode. The lipid library covers diverse lipid classes including fatty acids, acylcarnitines, phosphatidic acids (PAs), phosphatidylcholines (PCs), phosphatidylethanolamines (PEs), phosphatidylserines (PSs), phosphatidylglycerols (PGs), sphingomyelins (SMs), lysophospholipids (LysoPA, LysoPC, LysoPE, LysoPS, and LysoPI), ceramides, glucosylceramides, and mono-, di-, and triacylglycerol lipids (MG, DG, and TG, respectively).

The nonlipid small-molecule library was generated from experimental CID MS/MS spectra from proton or sodium adducts and deprotonated ions corresponding to metabolites commonly found in human biofluids, such as urine and blood serum or plasma deposited in MassBank^12^ and GNPS^13^ databases (additional information regarding the spectra sources are given in the Supporting Information). In contrast to lipids, the structural diversity and resulting variety of MS/MS fragmentation patterns of small molecules complicates the attribution of experimental fragment *m*/*z* values to the corresponding fragment ion formulas and structures. Therefore, the experimental MS/MS *m*/*z* values were used for the nonlipid libraries. A total of 179 small-molecule entries covering positive and negative mode fragment ions have been included in the library. More details about the construction of the libraries can be found in the Supporting Information. In summary, the libraries can be considered in four groups: lipids positive, lipids negative, nonlipid metabolites positive, and nonlipid metabolites negative.

### Datasets for Workflow Development and Testing

For development and testing of the annotation workflow, we used human serum LC–MS AIF datasets from the Multi-Ethnic Study of Atherosclerosis (MESA).^27,28^ Human serum samples were analyzed on a Waters Acquity UPLC system connected to a Waters Xevo-G2 Q-ToF system operated in the MS^E^ mode(see Supporting Information). Three datasets were used: two lipidomics datasets (C8 reverse-phase (RP) chromatography acquired in electrospray (ESI) positive and negative polarities: Lipid+ and Lipid−) and a polar extract acquired using hydrophilic interaction chromatography in the ESI positive mode (HILIC+). Annotations were made by manual inspection of MS^E^ and MS/MS spectra on selected samples and matching accurate mass, isotope patterns, and fragmentation spectra to databases such as LipidMaps,^29^ Human Metabolome Database (HMDB),^30^ and MassBank.^12^ Manual annotations were mostly of confidence level 2 according to the Metabolomics Standards Initiative (MSI),^31^ except for some lipids in the positive mode RP and HILIC. In the latter cases, annotations (PC, PE, PS, PG, PA, DG, and TG) where the chain lengths could not be determined with the available MS/MS information were regarded as MSI confidence level 3 (Supporting Information).

The performance of the annotation workflow was tested on four additional datasets on different matrices and instruments. (a) Two datasets acquired in-house of hydrophilic extracts from adipose tissue (AT). Samples were analyzed by HILIC UPLC-MS using a Waters Acquity UPLC system connected to a Waters Synapt Q-TOF system in the MS^E^ mode in both ESI positive and negative polarities (see the Supporting Information).^32^ Annotations were made by manual inspection of MS^E^ and MS/MS spectra to confidence levels 2 and 3 as described above for MESA datasets. (b) Two publicly available datasets from the MetaboLights repository,^33^ corresponding to amniotic fluid (MTBLS666, level 2 and 3 annotations) and urine (MTBLS816, mostly level 1 annotations). These studies were selected to cover additional sample matrices. The annotations obtained using MetaboAnnotatoR were compared with those reported by the authors. Performance was summarized using precision and recall calculated from the number of correctly and incorrectly annotated and unannotated metabolite features (eqs 2 and 3)
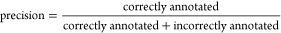
2
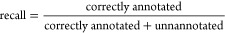
3

### Data Conversion and Processing

Raw LC–MS chromatograms were converted either to netCDF using Databridge software (Waters, Milford, MA, USA) or to mzML using Proteowizard’s msconvert version 3.0.^34^ A random selection of 100 LC–MS AIF chromatograms from both Lipid+ and Lipid– as well as HILIC+ datasets from the MESA cohort was processed in R using XCMS^23^ version 3.4.4 and RAMClustR package^5^ to obtain pseudo-MS/MS spectra (dataset-wide reconstruction) (see the Supporting Information).

### Automated Annotations Using MetaboAnnotatoR

Automated annotations were obtained for manually annotated features in the MESA and validation datasets. The automated annotations were obtained separately using the lipid and nonlipid metabolite libraries and the results were combined. MZ and matching weights (eq 1) were both set to *w*_mz_ = *w*_ma_ = 0.5. This value gave an adequate proportion of true positive annotations in preliminary tests using the MESA Lipid+ dataset (Supporting Information Figure S1) and should provide a reasonable scoring weight when all features are unknown hence annotation agreement cannot be assessed. Although the workflow contains the option to use retention time information of expected metabolite classes, this option was not used, to reflect real-life scenarios, where this information is not known.

### LC–MS Annotations Using MS-DIAL

Performance was compared to MS-DIAL^7^ (version 4.38) which is an established software package for automated pseudo-MS/MS reconstruction and annotation of AIF and other MS datasets. In MS-DIAL, mzML files were input and the default parameters for AIF analysis were used. These included minimum peak height of 1000; *m*/*z* search tolerance 0.01 Da for both MS1 and AIF scans; and pseudo-MS/MS deconvolution for single chromatogram using MS2Dec algorithm.^7^ The expected adducts were set to the same as those in MetaboAnnotatoR’s libraries. The default lipidomics databases (LipidBlast^35^) containing over 1 million spectra, and the full public MS/MS databases (V15), containing 327,763 MS/MS spectra, both obtained from MS-DIAL website (http://prime.psc.riken.jp/compms/msdial/) were used for spectral matching. Results from both databases were combined into a single table.

## Results and Discussion

### Typical Output of the Automated Annotation Pipeline

**Figure 2:**
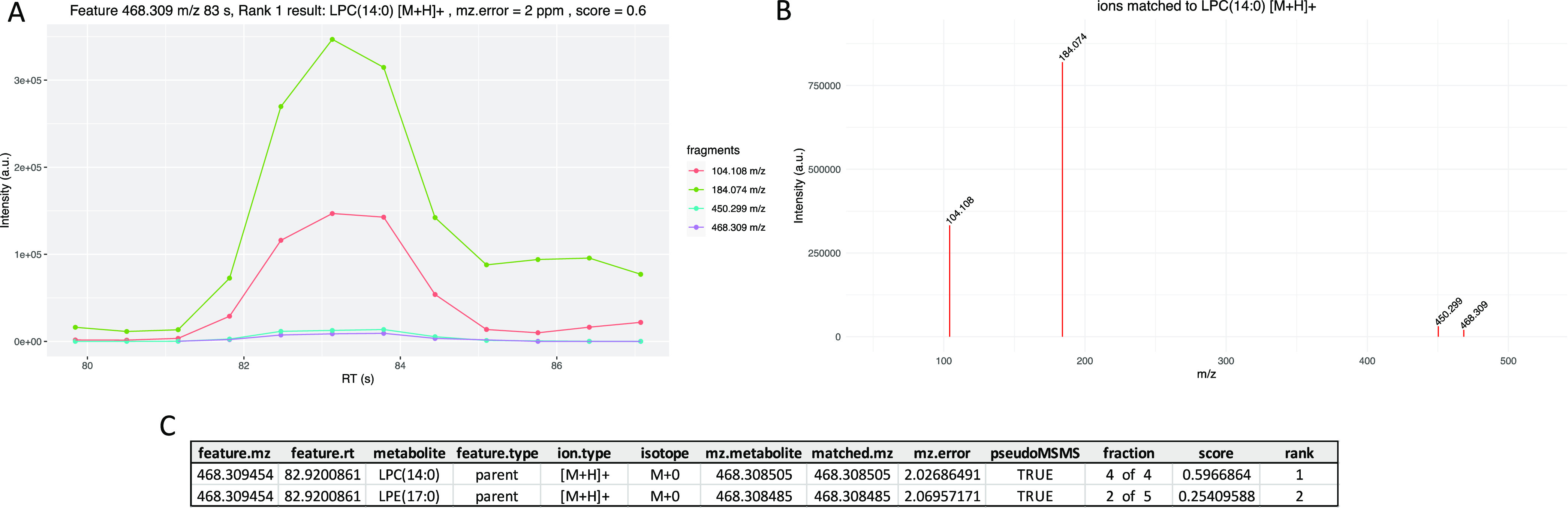
Automated annotation using MetaboAnnotatoR of feature 468.309 *m*/*z* 83 s from a representative sample of the MESA human serum Lipid+ dataset: (A) matched EICs and (B) corresponding pseudo-MS/MS spectrum of ions matched for the rank 1 candidate. (C) Table with ranked candidates for the same feature. Legend: mz.error—*m*/*z* error, *E*, in ppm; mz.metabolite—*m*/*z* of the parent ion of the matched candidate; matched.mz—*m*/*z* of the matched parent or fragment; fraction—number fragments of each candidate that have been matched to the target pseudo-MS/MS; and pseudo-MS/MS—logical value indicating if a pseudo-MS/MS was obtained (TRUE) or not (FALSE).

Representative outputs based on a single sample are shown in Figure 2. The matched ions for the rank 1 annotation are illustrated by overlapped EICs, which reveal the retention time agreement between the fragments of the candidate (Figure 2A). The matched ions are shown in a pseudo-MS/MS spectrum (Figure 2B). The graphical results of each annotation are accompanied by a table containing the ranked candidates for the target feature (Figure 2C), which provides information on the type of feature (parent or fragment), adduct, and isotope. Additionally, the *m*/*z* error between the feature and candidate is shown, as well as the number fragments of each candidate that have been matched to pseudo-MS/MS (fraction). The outputs when using RAMClustR deconvolved pseudo-MS/MS are equivalent except that EICs for the matched features are not shown (Supporting Information Figure S2). The outputs from Figure 2 allow the analyst to evaluate the quality of the annotation and decide on its confidence.

### Performance Evaluation: Comparison with Manual Annotations

To evaluate the performance of the automated annotation workflow, a comparison was made with annotations from manual assessment of AIF, MS/MS, accurate mass, and isotopic patterns. A total of 599 manually annotated features were obtained from human serum datasets from the MESA cohort, namely, Lipid+ (192 features), Lipid– (147 features), and HILIC+ (260 features) (Supporting Information). These annotations were then compared to the corresponding automated annotations obtained from (1) a representative quality control (QC) sample and (2) a RAMClustR (RC) object generated from 100 samples per dataset (see the Supporting Information). The features corresponded to different types of adducts, isotopologues, and in-source fragments from both lipid and nonlipid metabolites (Figures S3 and S4). The features used in this comparison correspond to all manually annotated features available at the time of analysis and include adduct entries that might not be found in the fragment libraries and thus cannot be correctly annotated by the workflow (e.g., [2M + Na]^+^ or [M + 2H_2_PO_4_Na]^−^). This allowed a more realistic evaluation of the annotation performance.

The number of automatically annotated features in agreement or disagreement with the manual annotations as well as those for which no annotation was obtained (no database match) is summarized in Table 1. Most of the features are correctly annotated at rank 1 (highest score) in all datasets.

**Table 1:** Comparison between Automated and Manual Annotations of MESA Datasets^a^

	number of correct annotations at each rank								
dataset	rank 1	rank 2	rank 3	rank 4	rank 5	incorrect	not annotated	precision (%)	recall (%)
Lipid+ QC^a^*N* = 192	134 (69.8%)	12 (6.3%)	4 (2.1%)	1 (0.5%)	1 (0.5%)	14 (7.3%)	26 (13.5%)	91.6	85.4
Lipid+ RC *N* = 192	134 (69.8%)	7 (3.6%)	5 (2.6%)	4 (2.1)	1 (0.5%)	17 (8.9%)	24 (12.5%)	89.9	86.3
Lipid- QC *N* = 147	75 (50.7%)	19 (12.8%)	2 (1.4%)	1 (0.7%)		10 (6.8%)	40 (27.1%)	90.6	70.8
Lipid– RC *N* = 147	92 (62.2%)	19 (12.8%)	2 (1.4%)	0 (0%)	1 (0.7%)	9 (6.1%)	24 (16.2%)	92.9	82.6
HILIC+ QC *N* = 260	143 (55.0%)	14 (5.4%)	6 (2.3%)		3 (1.2%)	31 (11.9%)	63 (24.2%)	84.3	72.5
HILIC+ RC *N* = 260	131 (50.4%)	23 (8.8%)	7 (2.7%)	6 (2.3%)	14 (5.4%)	39 (15.0%)	40 (15.4%)	82.3	81.9

a The QC sample corresponds to a pool of study samples. The RC (RAMClustR) object contains pseudo-MS/MS spectra arranged into clusters from the 100 study samples from the three datasets using XCMS and RAMClustR. Results are organized according to the rank where the correct annotation was found after ranking the annotation scores in the descending order. For precision and recall, an annotation was defined as correct if it was found in ranks 1–5.

The highest percentage of correct annotations in rank 1 was obtained for the Lipid+ datasets with around 70% accuracy or close to 80% when ranks 1 to 5 are combined. The accuracy for Lipid– and HILIC+ datasets was just above 50% for rank 1 and up to around 65% or 70–77% when ranks 1–5 are combined for the QC sample and RC object, respectively. For Lipid– (QC sample), no correct annotations were found above rank 4.

**Figure 3:**
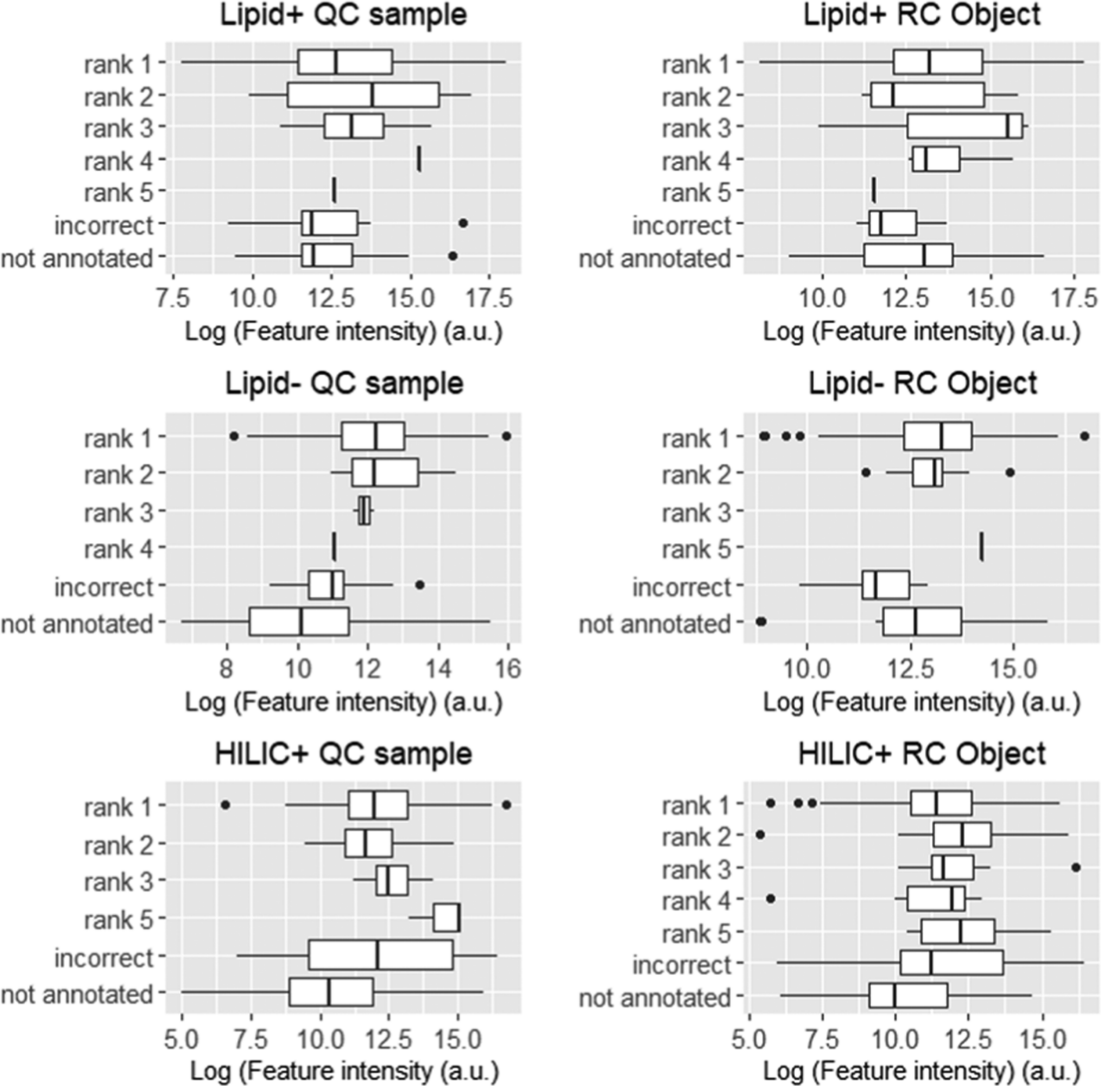
Relationship between annotation accuracy and feature intensity. Box plots show distribution of feature intensities for each rank of correct annotation, incorrectness, or the absence of annotation. Nondetected features (below min intensity) are not presented.

A smaller percentage of the features was not annotated in any of the datasets: 14% and 13% in Lipid+, 27% and 16% in Lipid–, and 24% and 15% in HILIC+ dataset for the QC samples and RC object, respectively. There was also a small percentage of incorrectly annotated features detected in all datasets: 7% and 9% for Lipid+, 7% and 6% for Lipid–, and 12% and 15% for HILIC+ for the QC sample and RC object, respectively. Despite these results, moderate-to-good precision can be claimed for the annotation workflow, with values just above 90% for the lipid datasets and slightly lower for HILIC datasets. Several factors can lead to not annotated or incorrectly annotated features. The intensity of the parent will strongly impact detection of the corresponding fragments. We investigated the relationship between feature intensity and annotation rank, incorrectness, and the absence of annotation (Figure 3).

In all datasets, rank 1 annotations span a wide range of intensities with higher median intensity values (Mann–Whitney *p* < 0.05) when compared to not annotated features, except for Lipid+ dataset. In the lipid datasets, rank 1 annotations had higher median intensities than incorrectly annotated features only in the Lipid– dataset (Mann–Whitney *p* < 0.05). This illustrates that feature intensity plays a role in whether a feature is annotated and in the Lipid–, if it is correctly annotated.

Two other factors that could give rise to incorrect and missing annotations are the absence of the true candidates from the libraries and the incorrect attribution of feature isotopologue. The latter will have an effect on the selection of candidate metabolites from the fragment libraries. These factors are investigated for the HILIC+ results in Figure S5A,B (Supporting Information).

Missed annotations for the high-intensity features of HILIC+ can be explained in part by the wrong attribution of the isotopologue (Figure S5B top) rather than missing candidates from the libraries (Figure S5A top). This was not observed for the incorrectly annotated features (Figure S5A,B bottom).

Features could also be incorrectly annotated if the wrong fragments are matched or if the fragments were missing because they were not detected. For HILIC, features with high intensity that were incorrectly annotated occurred at RTs with high ion density (around 240 s and close to 300 s, Figure S5C). This may have led to a higher degree of cross-compound fragment matching resulting in incorrect annotations.

### Evaluation of Annotation Performance: Comparison with MS-DIAL

**Figure 4:**
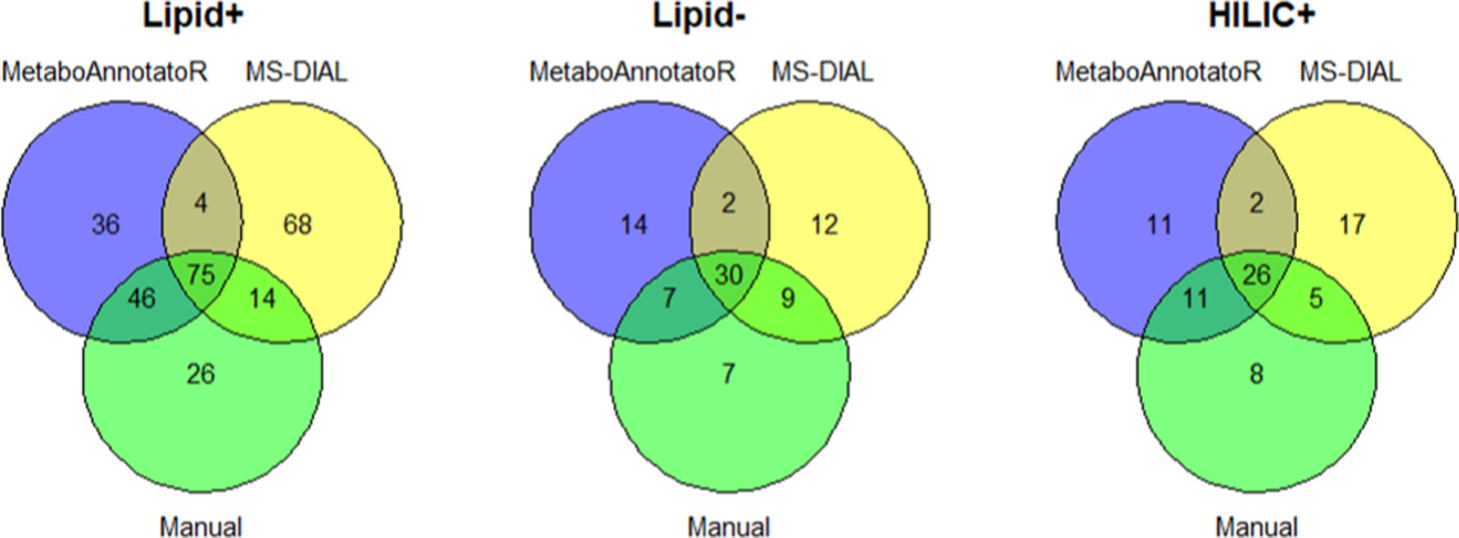
Venn diagrams showing the number of overlapped annotations between manually annotated features and those from MetaboAnnotatoR and MS-DIAL for Lipid+ (*n* = 161 features), Lipid– (*n* = 53 features), and HILIC+ (*n* = 50 features).

The automated annotation workflow was tested against another software package for LC–MS analysis, MS-DIAL.^7^ This performs automated processing, deconvolution, and annotation of AIF data (among other tandem-MS modalities) and uses lipid and other metabolite libraries for spectral matching. Therefore, it seemed the most appropriate software against which to compare our workflow. For this purpose, the same AIF QC datasets used for comparison against the manual annotations were processed in MS-DIAL using MS2Dec deconvolution algorithm^7^ and matched against the software lipid and metabolite MS/MS spectra libraries. Since MS-DIAL only considers M + 0 isotopologues for further annotation, the comparison was made using features corresponding to this isotopologue. Only features annotated by MS-DIAL and MetaboAnnnotatoR and those manually annotated were compared. This corresponded to 161 features for Lipid+, 53 for Lipid–, and 50 for HILIC+ observed in the representative QC sample. For MetaboAnnotatoR, only rank 1 annotations were used, to make a fairer comparison against MS-DIAL, which only yields top-ranked matches as annotation results. Annotations are provided in the Supporting Information. All datasets showed good agreement between manual and automated annotations with overlaps above 50% (Figure 4).

We observed that MetaboAnnotatoR achieved a better overlap with manual annotations than MS-DIAL for Lipid+ [121/161 (75%) *vs* 89/161 (55%)] and HILIC+ [37/50 (74%) *vs* 31/50 (62%) (Figure 4)]. The reason for the slightly superior accuracy of MetaboAnnotatoR could be a better recognition of features corresponding to [M + Na]^+^ and [M + K]^+^ adducts than MS-DIAL, particularly for common lipid classes such as PCs, LPCs, SMs, and TGs (Supporting Information). In fact, the default lipid MS/MS spectral library used by MS-DIAL, LipidBlast, does not include [M + K]^+^ adducts.^35^ Nevertheless, MS-DIAL can detect many types of adducts in MS1 analysis. However, this adduct information might have not been taken into account at the spectral matching between the pseudo-MS/MS and database MS/MS spectra, which resulted in incorrect annotations. For Lipid–, differences in overlap between manual and automated annotations were less pronounced with 37/53 (70%) for MetaboAnnotatoR and 39/53 (74%) for MS-DIAL. There were also a few instances in all datasets where MetaboAnnotatoR and MS-DIAL both picked the same incorrect annotations (overlap between MetaboAnnotatoR and MS-DIAL, Figure 4).

Overall, these results demonstrate that the workflow implemented in MetaboAnnotatoR can outperform the well-established software MS-DIAL in LC–MS AIF annotation. The workflow has another practical advantage over MS-DIAL, which is the possibility for the user to choose the features to be annotated. These can be, for instance, those that have a statistical significance in a study. In MS-DIAL, such a selection is currently not possible, and the annotations are performed in an untargeted manner. This increases the runtime, which can take several hours for one sample, with no guarantee that the desired features will be peak-picked and annotated. MetaboAnnotatoR can take from 1 s or less (RAMClusteR objects) to 2 min (raw chromatogram) per feature. The user can focus on features of interest in specific samples, for instance, where the features are found in high abundance.

A key factor that needs some consideration when comparing the two software packages is the reference libraries. There are considerable differences in the numbers of entries between both approaches: over 327,763 MS/MS records in MS-DIAL for several metabolite classes (publicly available spectra) and over 1 million Lipid MS/MS entries compared to 90,425 Lipid and 179 small-molecule metabolite entries for MetaboAnnotatoR. An almost complete overlap of lipid classes and adducts between MS-DIAL’s libraries and those of MetaboAnnotatoR would be expected. A comprehensive evaluation of such an overlap was not performed given the complexity and size of the libraries. Nevertheless, the lower number of entries in MetaboAnnotatoR could also explain in part the somewhat better false-positive rate for some features in comparison with MS-DIAL.

### Application to Other Datasets

The performance of the workflow was tested on four additional datasets from different species, sample types, and instrument combinations. Comparisons were made against manual annotations (adipose tissue datasets) or those reported by the study authors (MetaboLights datasets). The results are summarized in Table 2. The precision of the automated workflow is generally good (>80%), with most correct annotations being captured at rank 1. The exception is the amniotic fluid dataset which had a modest precision (60%). The recall results are excellent (>80%) for the adipose tissue datasets, where most features correspond to lipids, but low for the two MetaboLights datasets. This results from the high number of false-negatives (no match to a database entry) for nonlipid metabolites, which highlights the need for large libraries. Detailed results can be found in the Supporting Information.

**Table 2:** Automated Annotation of Additional Studies^a^

study/dataset	species	sample	chromatography/MS instrument	annotations correct/reported	precision (%)	recall (%)	no ref.
AT+	*Homo sapiens*	adipose tissue	HILIC/Waters Synapt Q-ToF, ESI^+^	26/37	81.3	83.9	2 (5.4%)
AT–	*Homo sapiens*	adipose tissue	HILIC/Waters Synapt Q-ToF, ESI^–^	16/21	84.2	88.9	2 (9.5%)
MTBLS666	*Sus scrofa*	amniotic fluid	RP-C18/Waters Synapt Q-ToF, ESI^–^	3/32	60.0	10.0	27 (84.4%)
MTBLS816	*Homo sapiens*	urine	HILIC Positive/Agilent Q-ToF, ESI^+^	31/107	88.6	30.1	60 (56.1%)

a Adipose tissue extract (AT) HILIC datasets and two publicly available datasets from the MetaboLights repository (MTBLS). Precision and recall refer to rank 1 automated annotations. No ref.—Annotations missed due to the absence of reference from the library. Annotations reported by the studies were level 2 and 3 except for MTBLS816 which is mostly composed of level 1 annotations (105 of 107).

## Conclusions

We have described a novel workflow and software package, MetaboAnnotatoR, to automatically annotate features from metabolomics and lipidomics untargeted LC–MS experiments acquired using AIF.

Existing software packages rely on databases of MS/MS spectra and the majority focus on lipidomics. In contrast, MetaboAnnotatoR can be applied to both lipids and other metabolites. Its libraries are composed solely of fragment ion information, without experimental MS/MS spectra of pure standards, a strategy which has been used in LipidMatch^19^ and R-Metabolist^9,20^ R packages, for lipids and nonlipids, respectively. This has the advantage that users can input fragment information even when MS/MS spectra are not available in a spectral database, for example, reference spectra only existing in the literature. In our workflow, we implemented a novel scoring system based on individual fragment occurrence scores which allows us to compensate the absence of relative intensities (as in an experimental MS/MS spectrum). This also obviates variations in intensity from MS/MS spectra acquired in different instruments with different fragmentation energies. Another unique feature is the ability for peaks not captured by pseudo-MS/MS to be searched in AIF scans, which can compensate for co-elution of molecules producing commonly shared fragments. Contrary to other software such as MS-DIAL, MetaboAnnotatoR is able to annotate the different isotopologues from isotopic series, as well as in-source fragments. This enables the direct application of the workflow to features obtained from XCMS or similar software. The main weakness of MetaboAnnotatoR as compared with other approaches is the relatively small library size (particularly nonlipids) compared to other approaches such as MS-DIAL. However, these can be customized by the user, for instance, by importing external libraries as .txt and .msp files. The default libraries will be expanded in future versions of the software.

Using default databases and options, good precision (82–92%) and recall (82–85%) values were obtained for human serum datasets for high-rank scores (1–5). These results equal or outperform those obtained using MS-DIAL, the state-of-the-art software for AIF data annotation. Further validation was obtained for other biological matrices and different types of instruments. These showed modest-to-good (60–88%) precision but low recall due to the small size of the fragment libraries, in particular, the nonlipid library.

The annotation of AIF datasets acquired on higher mass accuracy MS instruments (e.g., Orbitraps), although not tested here, is also possible using MetaboAnnotatoR. This should lead to equally good or even better annotations results. Although only AIF is currently supported, future developments might expand MetaboAnnotatoR’s application to other DIA schemes, such as SWATH.

We believe this workflow addresses a key need for more effective annotation of untargeted LC–MS AIF data and will be of value in many metabolomics and lipidomics applications.

## Supporting Information Available

Small-molecule library sources (XLSX)
Serum MESA annotations (XLSX)
Annotation comparisons with MS-DIAL (XLSX)
Annotations for the additional datasets (XLSX)
Additional experimental details and supplementary figures (PDF)
