# Supplementary material for: Automated Annotation of Untargeted All-Ion Fragmentation LC–MS Metabolomics Data with MetaboAnnotatoR
Source: Anal Chem. 2022 Feb 18;94(8):3446–55. doi: 10.1021/acs.analchem.1c03032 (PMC8892435; doi:10.1021/acs.analchem.1c03032)
Supplement: Supplementary file 5 — ac1c03032_si_005.pdf [file ac1c03032_si_005.pdf]

Supplementary Information

Automated annotation of untargeted all-ion fragmentation LC-MS metabolomics data with MetaboAnnotatoR

Gonçalo Graça,[*,†] Yuheng Cai,[†] Chung-Ho E. Lau,[‡] Panagiotis A. Vorkas,[§,‖] Matthew R. Lewis,[⊥] Elizabeth J. Want,[§] David Herrington,[#] and Timothy M. D. Ebbels[*,†]

[†] Section of Bioinformatics, Division of Systems Medicine, Department of Metabolism, Digestion and Reproduction, Imperial College London, South Kensington Campus, Sir Alexander Fleming Building, London SW7 2AZ, UK

[‡] Department of Epidemiology and Biostatistics, School of Public Health, Imperial College London, London W2 1PG, UK

[§] Section of Systems Medicine, Division of Systems Medicine, Department of Metabolism, Digestion and Reproduction, Imperial College London, South Kensington Campus, Sir Alexander Fleming Building, London SW7 2AZ, UK

[‖] Institute of Applied Biosciences, Centre for Research and Technology Hellas, 57001 Thessaloniki, Greece

[⊥] Section of Bioanalytical Chemistry and National Phenome Centre, Division of Systems Medicine, Department of Metabolism, Digestion and Reproduction, Imperial College London, Hammersmith Campus, IRDB Building, London W12 0NN, UK

[#] Section on Cardiovascular Medicine, Wake Forest School of Medicine, Winston-Salem, NC 27157, USA

* Corresponding authors
E-mail: g.gomes-da-graca@imperial.ac.uk; t.ebbels@imperial.ac.uk

Table of contents

Additional experimental details S3

MESA UPLC-MS datasets S3

Adipose tissue UPLC-MS datasets S4

Processing of MESA UPLC-MS datasets using XCMS and RAMClustR S5

Manual annotations of MESA and Adipose tissue samples S6

Construction of the metabolite fragment libraries S6

Supporting figures S7

Figure S1 – Agreement between manual and automated as a function of scoring weights S7

Figure S2 – Automated annotation results using RAMClustR object S8

Figure S3 - Chemical classes of the annotated metabolites in the MESA datasets S9

Figure S4 - Feature types of the annotated metabolites in the MESA datasets S10

Figure S5 – Effects of missing library candidates and wrongly determined isotopologues S11

References S12

Additional experimental details

MESA UPLC-MS datasets

These datasets consisted of LC-MS chromatograms acquired for 1977 human serum samples from the population study Multi-Ethnic Study of Atherosclerosis (MESA) study.[1,2] The serum samples were analysed by reverse phase (C8) ultra-performance liquid chromatography using gradient elution (Lipid+ and Lipid- datasets) as well as by hydrophilic interaction (HILIC) ultra-performance liquid chromatography (HILIC+ dataset). Prior to analysis, all samples were thawed, and serum protein precipitation was performed using cold isopropanol (Lipid datasets) or cold acetonitrile (HILIC dataset), incubated for 2 h at -20°C and centrifuged. A quality control sample (QC) resulting from a pooled mixture of all analysed samples was prepared for LC column equilibration and analytical drift correction (internal QC). Additionally, a commercial serum sample (external QC) and another serum sample unrelated to the study (Long term reference) were used to assess inter-batch variability. Each serum supernatant was analysed in an ACQUITY UPLC® system coupled to a Xevo G2-S ToF mass spectrometer (Waters, Milford, MA, USA). For the Lipid datasets, the samples were separated in a ACQUITY UPLC® BEH C8 1.7µm at 55°C. The mobile phases were composed of a solution of 5 mM Ammonium acetate + 0.05% Acetic acid in a mixture of 25:25:50 proportion of Isopropanol, acetonitrile, and ultra-pure water (Mobile phase A); and 5mM Ammonium acetate + 0.05% Acetic acid in a 50:50 mixture of Acetonitrile and Isopropanol (Mobile phase B). After injection of 10 µL sample, the chromatography was run at flow rate of 0.6 mL/min using the gradient: 99% A (0-2 min); 70% A (2-11.5 min) and 10% A (11.5-12 min).

The HILIC dataset was collected using the same UPLC instrumental setup using a 2.1 × 150 mm ACQUITY BEH HILIC column (Waters Corp., Milford, MA, USA) maintained at 40°C during analysis. The mobile phases used consisted of acetonitrile with 0.1% formic acid (50:50 mixture) (mobile phase A) and 20 mM ammonium formate in water with 0.1% formic acid (mobile phase B). The chromatographic separation occurred at 0.6 mL/min flow rate. After sample injection, a 0.1 min isocratic separation occurred at initial conditions (95% A). This was followed linear gradient between 95% and 80% A from 0.1 to 4.6 min. A more rapid gradient was then applied from 80% to 50% A between 4.6 min and 5.50 min. This was followed by an isocratic period between 5.50 and 7.00 min (50% A). The gradient conditions were changed to 95% A at 7.10 min and the flow rate was gradually increased to 1 mL/min until 12.50 min. After this time the flow rate was returned to 0.6 mL/min until 15 min to re-establish the initial conditions and enable the injection of a new sample.

For both RP-C8 and HILIC separations, the MS data was collected separately in positive and negative mode electrospray ionization. The capillary voltage was set to 1.5 kV for positive mode and 1.0 kV for

negative mode, cone voltage was 20 V, source temperature was set at 120 °C with a cone gas (nitrogen) flow rate of 50 L/h, a desolvation gas temperature of 600 °C, and a nebulization gas (nitrogen) flow of 1000 L/h. MS data was acquired in MS^E data acquisition mode in which MS scans are acquired by alternating all-ion fragmentation with no fragmentation.[3] Mass spectral data were collected in centroid mode using a mass range 50-2000 m/z for low-collision energy MS scans and 100-2000 m/z for high-collision energy MS scans for RP-C8 and 50-1200 m/z for HILIC low and high collision energy scans. When no fragmentation was employed (odd scans) a low collision energy (4 eV) was used and a high collision energy (ramp (10-30 eV) was used to acquire for fragmentation scans (even scans). Leucine enkephalin (2 ng/µL, 50% ACN, 0.1% FA) was used for lock mass correction which was infused at 20 µL/min. Lock mass data were collected every 60 s for 0.2 s.

The HILIC Negative was not used in this work due to the lower number of annotations available for this dataset. The LC-MS chromatograms were converted to netCDF format using Waters DataBridge software and, each function 1 (MS1 – low collision energy) was imported and processed using XCMS v. 3.1 to produce feature tables, from which the features were selected for manual and automated annotation.

Adipose tissue UPLC-MS datasets

A second experimental dataset used for the purpose of testing the MetaboAnnotatoR workflow consisted of human adipose tissue acquired by HILIC LC-MS and published elsewhere (Vorkas et al. 2018). Briefly, the tissues (~25 mg) were extracted using a 1:1 mixture of water/methanol assisted by zirconium beads, using a bead-beater. The extracts were centrifuged, and the supernatants were dried under vacuum. The dried samples were reconstituted in a mixture of acetonitrile (ACN)/water (95:5). Samples were vortex mixed, sonicated, and centrifuged and transferred to glass vials which were maintained at 4°C during analysis. Samples were analysed on an Acquity UPLC System (Waters Corp., USA), and 5 mL of each sample were injected in an Acquity UPLC BEH HILIC 2.1 × 100 mm, 1.8 µm, column (Waters Corp, USA) kept at 40°C, and separated by gradient elution. The mobile phases consisted of solutions of mobile phase A: acetonitrile (ACN)/water (95:5), 10 mM ammonium acetate, and 0.1% formic acid; mobile phase B: ACN/water (50:50), 10 mM ammonium acetate, and 0.1% formic acid. The elution gradient and flow rate were set as follows: 99% A (0.0–2.0 min; 0.4 mL/min), 99–45% A (2.0–8.0 min; 0.4 mL/min), 45–1% A (8.0–9.0 min; 0.4 mL/min), 1% A (9.0–9.1 min; 0.4–0.6 mL/min), 1% A (9.1–11.0 min; 0.6 mL/min), 1–99% A (11.0–11.1 min; 0.6 mL/min), 99% A (11.1–17.0 min; 0.6 mL/min), 99% A (17.0–17.1 min; 0.6–0.4 mL/min) and 99% A (17.1–21.0 min;0.4 mL/min). The MS data was acquired in a Waters Synapt Q-ToF. The mass range was set between 50 to 1200

m/z. The MS^E mode (continuum mode) was used for MS acquisition. Three functions (parallel acquisition channels) were employed for acquisition: function 1, low collision energy; function 2, high collision energy; and function 3, lock mass acquisition channel. For both low and high collision energy functions, survey scan time was set to 0.2 s. For high collision energy acquisition, collision energy was ramped for low masses 20–40 V and linearly increased up to a 30–50 V ramp, for high masses. Leucine enkephalin (2 ng/µL, 50% ACN, 0.1% FA) was used for lock mass correction. Lock mass data were collected every 30 s for 0.2 s. Other MS parameters were set as follows: cone voltage 30 V, capillary voltage 2 KV, source temperature 120 °C, desolvation temperature 550 °C, and desolvation gas 900 L/h. Samples were acquired in positive and negative ionisation modes (separately), making a total of two different datasets: HILIC Positive and HILIC Negative. The LC-MS chromatograms acquired in continuum mode were centroided using Waters MassLynx software v. 4.1 and then converted to netCDF format using Waters DataBridge software, prior to automated annotation MetaboAnnotatoR. Features were annotated manually using MS/MS acquired using data-dependent acquisition and MS^E acquisition.

Processing of MESA UPLC-MS datasets using XCMS and RAMClustR

From the three MESA LC-MS datasets (Lipid Positve, Lipid Negative and HILIC Positive) 100 samples were selected randomly (no QCs included). The raw chromatographic data was converted to netCDF format using DataBridge software (Waters), which separated the no-collision and high-collision energy scans into separate files (two files per sample MS1 & AIF, 200 files per MESA dataset). These files were all imported and processed in XCMS using the parameters summarised in Table S1. Briefly, after importing the data files, the chromatograms from MS1 and AIF were peak picked using the centWave algorithm and dealt with as a single dataset. In order to reduce the computational time, the Lipid- and HILIC+ datasets, which were the larger in size compared to Lipid+, were peak picked using higher values for prefiltering (Table S1). Before non-linear RT alignment was performed, a first grouping was applied using the bandwidths detailed in Table S1. A second grouping was performed after RT correction. Finally, any missing peaks were gap-filled using the function “fillPeaks” with method = “chrom” option. Each of the three datasets were processed separately.

RAMClustR was run on each of the final XCMS processing object, using the function “ramclustR” after specifying the experimental metadata and indicating the tags for MS1 and AIF scans. The resulting RAMClustR object, which contained the clusters containing the groups related to the same parent ion (i.e., the deconvoluted pseudo-MS/MS) was used as input for annotations by MetaboAnnotatoR.

Table S1 – XCMS parameters used to process AIF data for RAMClustR deconvolution.

	XCMS parameters			
**Dataset**	**Peak-picking**	**First grouping**	**RT correction**	**Second grouping**
**Lipid Positive**	method = "centWave"<br>peakwidth = c(3,10)<br>ppm = 20<br>snthresh = 5<br>mzdiff = 0.01<br>prefilter = c(1,5)	method = "density"<br>minfrac = 0<br>minsamp = 0<br>bw = 2<br>mzwid = 0.01	smooth = "loess"<br>missing = 10<br>extra = 10<br>span = 10	bw = 2
**Lipid Negative**	method = "centWave"<br>peakwidth = c(2,25)<br>ppm = 20,<br>mzdiff = 0.01,<br>snthresh = 10,<br>noise = 300,<br>prefilter = c(5,350)	method = "density"<br>minfrac = 0.3<br>minsamp = 0<br>bw = 2<br>mzwid = 0.01	smooth="loess",<br>missing=15<br>extra=15<br>span=5	bw = 2
**HILIC Positive**	method = "centWave"<br>peakwidth = c(2,15)<br>ppm = 20<br>snthresh = 5<br>mzdiff = 0.01<br>prefilter = c(2,50)	method = "density"<br>minfrac = 0.3<br>minsamp = 0<br>bw = 2<br>mzwid = 0.01	smooth="loess",<br>missing=10<br>extra=10<br>span=5	bw = 2

Manual annotations of MESA and Adipose tissue samples

Features from the MESA and Adipose tissue datasets were annotated on samples where these features were found in higher abundances by manual inspection of the MS^E and MS/MS and matching accurate mass, isotope patterns and fragmentation spectra to databases such as LipidMaps, Human Metabolome Database (HMDB) and MassBank. Then these annotations were propagated to other samples, such as the QCs, which were used to test the automated annotation procedures by matching at the m/z of parent ion (and as many fragments as possible) and RT profiles. Manual annotations were mostly of confidence level 2 according to the Metabolomics Standards Initiative (MSI), except for some lipids in positive mode RP and HILIC. In the latter cases, annotations (PC, PE, PS, PG, PA, DG and TG) where the chain lengths could not be determined with the available MS/MS information were regarded as MSI confidence level 3 (Supplementary File 2).

Construction of the metabolite fragment libraries

Lipid libraries were adapted from the libraries contained in the LipidMatch R package, which contain the theoretical m/z of the fragments expected in CID spectra of lipids. These libraries were modified to retain only those fragments that are observed in experimental MS/MS. Occurrence scores were attributed to each fragment yielding implicit fragmentation rules: a score of 0.9 was divided equally between the fragments most observed experimentally, and in literature reported electrospray

ionisation (ESI) collision induced decay (CID) MS/MS experiments, whereas the remaining 0.1 was divided equally between the other fragments, so that the total score sums to 1.

For the non-lipid metabolites, due to their structural diversity, the experimental MS/MS spectra were used. These consisted of MS/MS spectra from MassBank and GNPS databases acquired on different types of instruments, from low resolution triple-quadrupoles to high resolution Fourier transform-type instruments, using a wide range of collision energies between 5 and 65 eV including collision energy ramps, as detailed on Supplementary File 1.

The occurrence score for each fragment was calculated considering the MS/MS peak relative intensities. A score of 0.9 was divided equally between peaks above 10% of the most intense peak. A score of 0.1 was divided equally between the peaks with relative intensities between 0.05% and 10%, and peaks below 0.05% relative intensity were considered noise. Custom libraries can be imported to MetaboannotatoR from .txt and .msp formats.

Supporting figures

Figure S1 – Agreement between manual and automated as a function of scoring weights

Using the MESA Lipid+ dataset, the matching weight parameter was changed from 0.5 to 1 and its effect on the agreement between manual and automated annotations provided by MetaboAnnotatoR was inspected (Fig. S1).

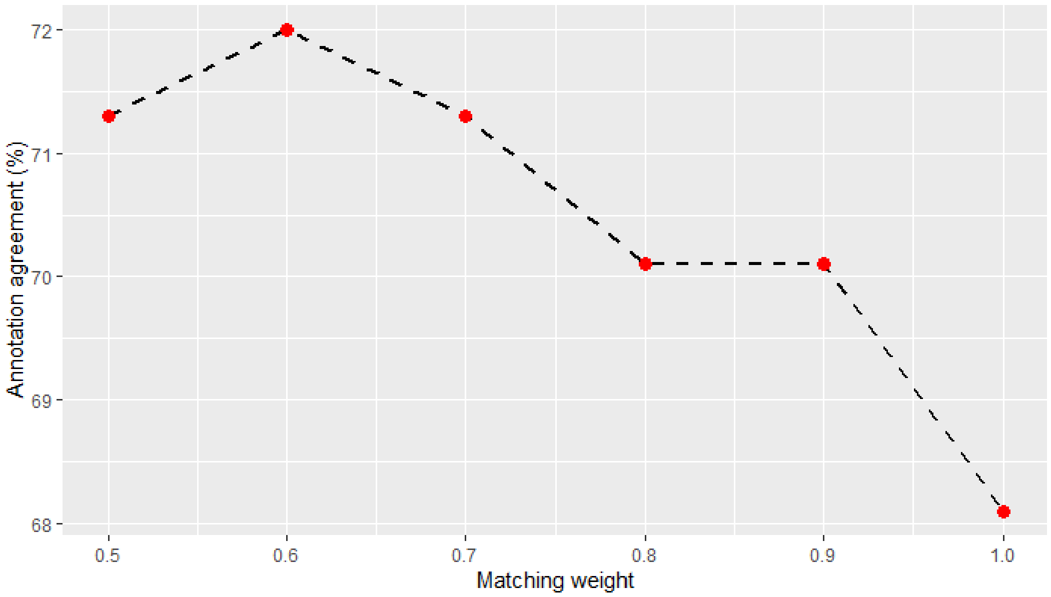

Fig. S1 – Percentage of agreement between manual annotations and rank 1 annotations as function of fragment matching weight illustrated for a quality control (QC) sample MESA Lipid+ dataset.

The value of matching weight of 0.5 was considered to be adequate for the dataset and was applied throughout the automated annotations provided in the text.

A typical annotation output result of feature annotation using RAMClustR pseudo-MS/MS objects is shown in Fig S2. No EICs are obtained this graphical report because the RAMClustR object only contains processed data and EICs can only be obtained from the raw chromatograms.

Figure S2 – Automated annotation results using RAMClustR object

A

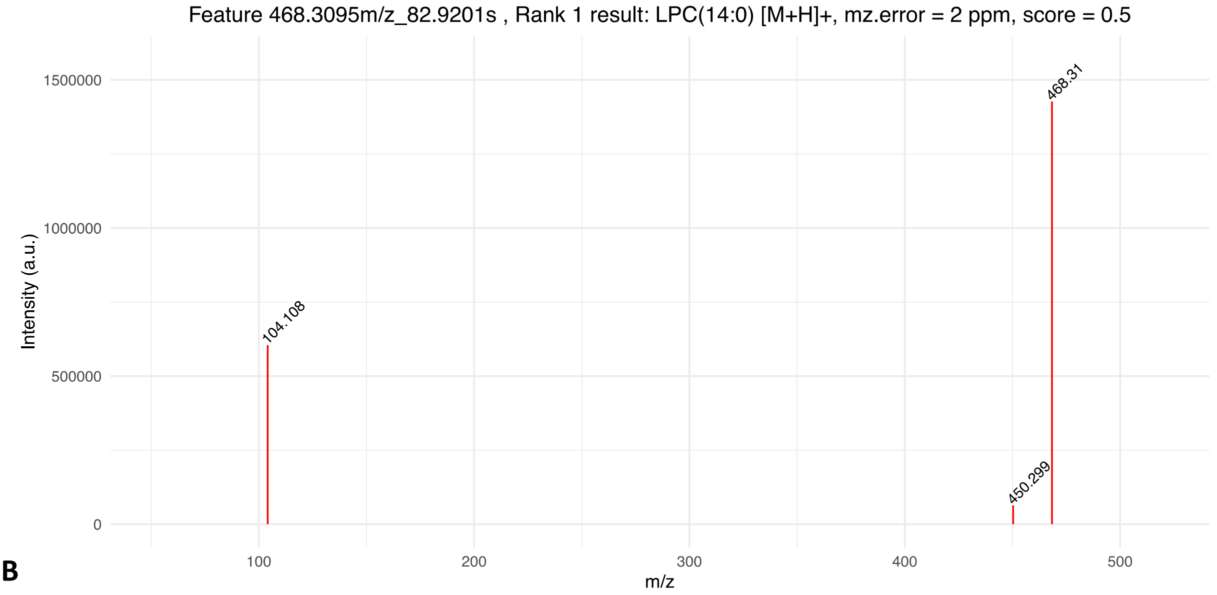

B

feature.mz	feature.rt	metabolite	feature.type	ion.type	isotope	mz.metabolite	matched.mz	mz.error	pseudoMSMS	fraction	score	rank
468.309454	82.9200861	LPC(14:0)	parent	[M+H]+	M+0	468.308505	468.308505	2.02686491	TRUE	3 of 4	0.4966864	1
468.309454	82.9200861	LPE(17:0)	parent	[M+H]+	M+0	468.308485	468.308485	2.06957171	TRUE	2 of 5	0.26034588	2

Figure S2 - Results for the automated annotation of feature 468.309 m/z 83 s from the RAMClustR R object obtained for MESA human serum Lipid Positive dataset: A) Matched pseudo-MS/MS spectrum for the rank 1 candidate. B) Table with ranked candidates for the same feature. Legend: mz.error - MZerror in ppm; mz.metabolite - m/z of the precursor ion of the matched candidate from the library; matched.mz – m/z of the library precursor or fragment matched to the target feature; fraction – number fragments of each candidate that have been matched to the target feature pseudo-MS/MS; pseudoMSMS – logical value indicating if a pseudo-MS/MS was obtained (TRUE) or not (FALSE).

Figures S3 and S4, summarise the metabolite chemical classes and feature types, respectively, that were tested in the MESA serum datasets.

Figure S3 - Chemical classes of the annotated metabolites in the MESA datasets

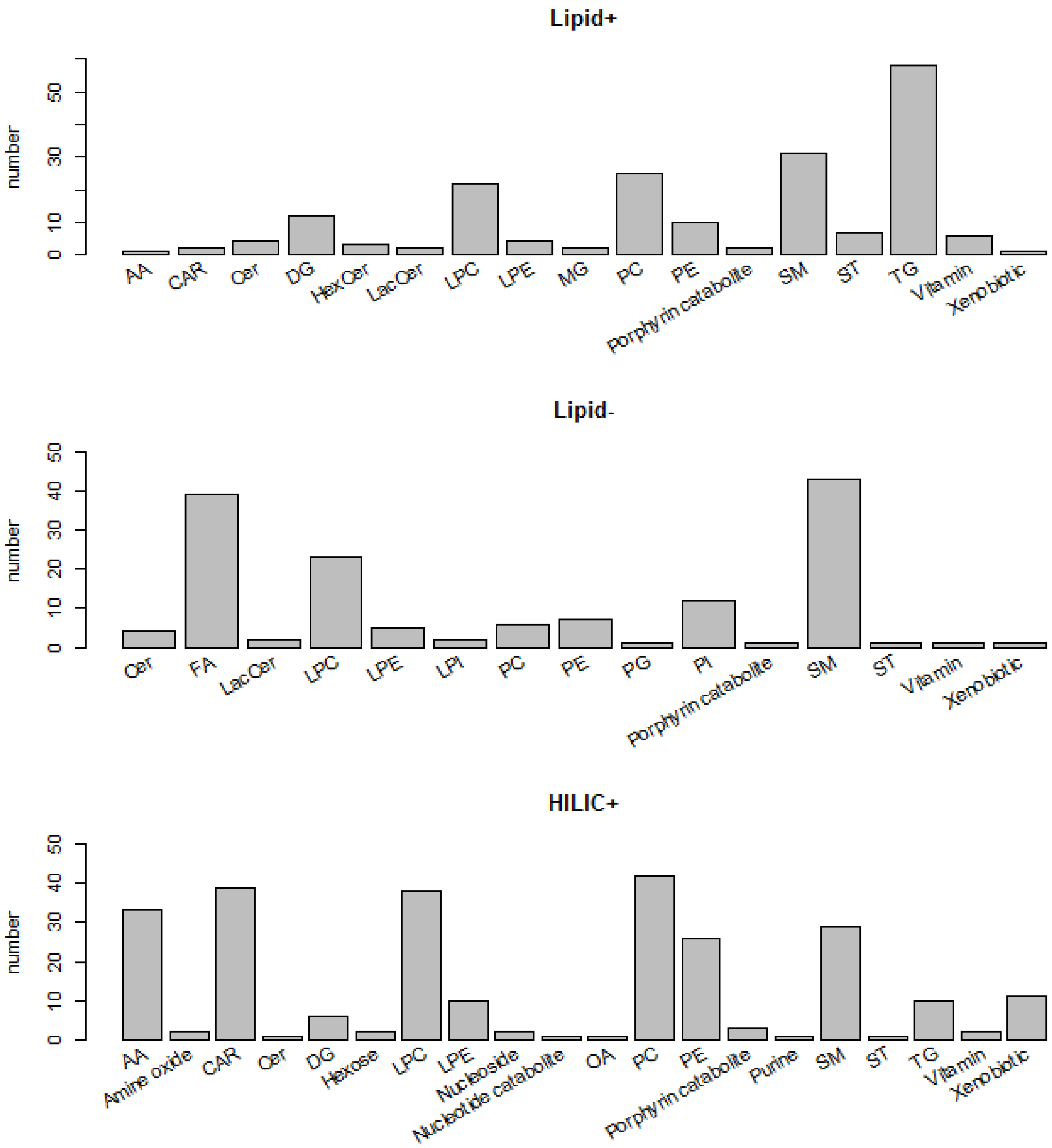

Figure S3 – Number of manually annotated metabolite features in MESA datasets in each metabolite class. Legend: AA, amino acids; CAR, carnitines; Cer, ceramides; DG, diglycerides; FA, fatty acids; HexCer, hexosylceramides; LacCer, lactosylceramides; LPC, lysophosphatidylcholines; LPE, lysophosphatidylethanolamines; LPI, lysophosphatidylinositols; MG, monoglycerides; OA, organic acids; PC, phosphatidylcholines; PE, phosphatidylethanolamines; PG, phosphatidylglycerols; PI, phosphatidylinositols; SM, sphingomyelins; ST, sterols (cholesterol, cholesterol esters and cortisol); TG, triglycerides; Vitamin, vitamins and vitamin metabolites.

Figure S4 - Feature types of the annotated metabolites in the MESA datasets

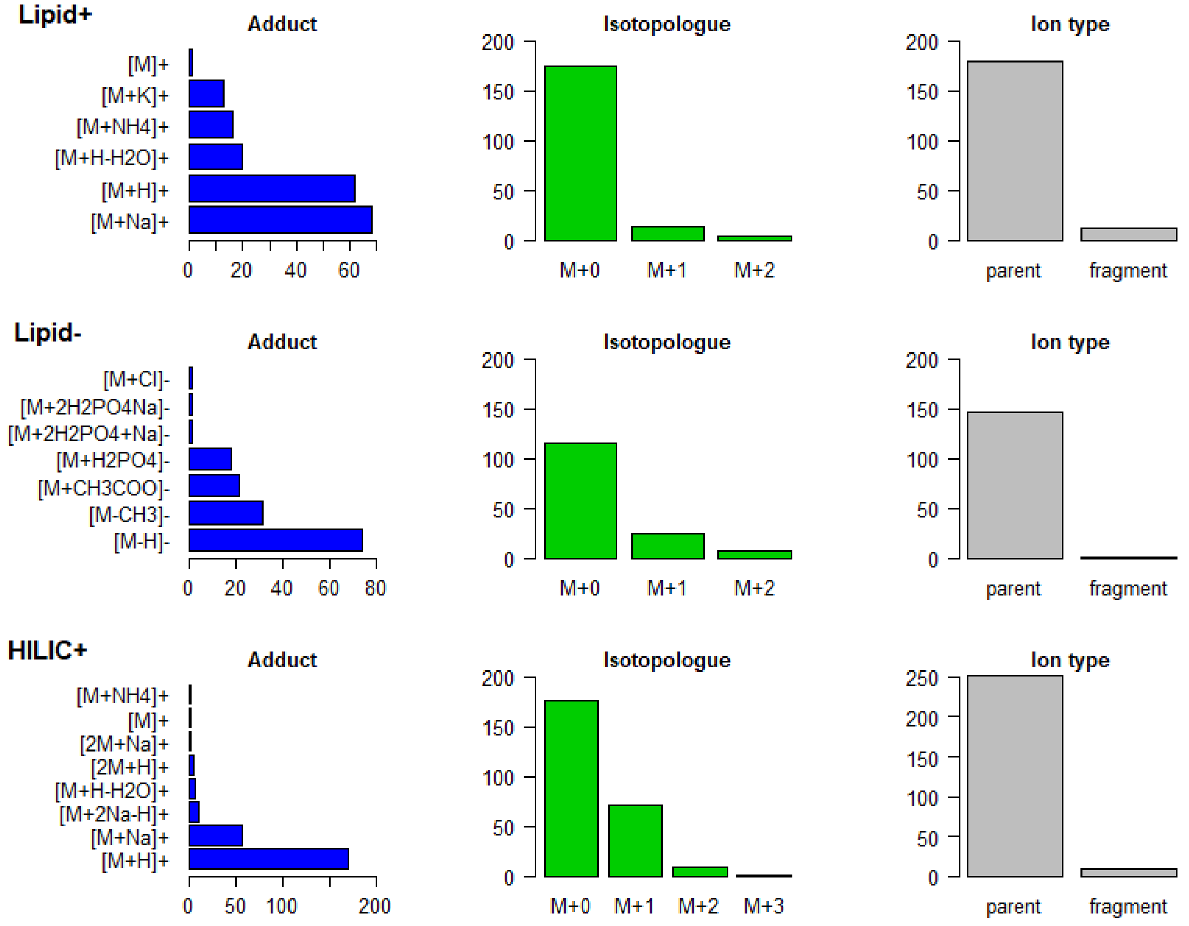

Figure S4 – Distribution of adducts, isotopologues and ion type (in-source fragment ion or parent ion) for the features tested in the MESA serum datasets.

Figure S5 – Effects of missing library candidates and wrongly determined isotopologues

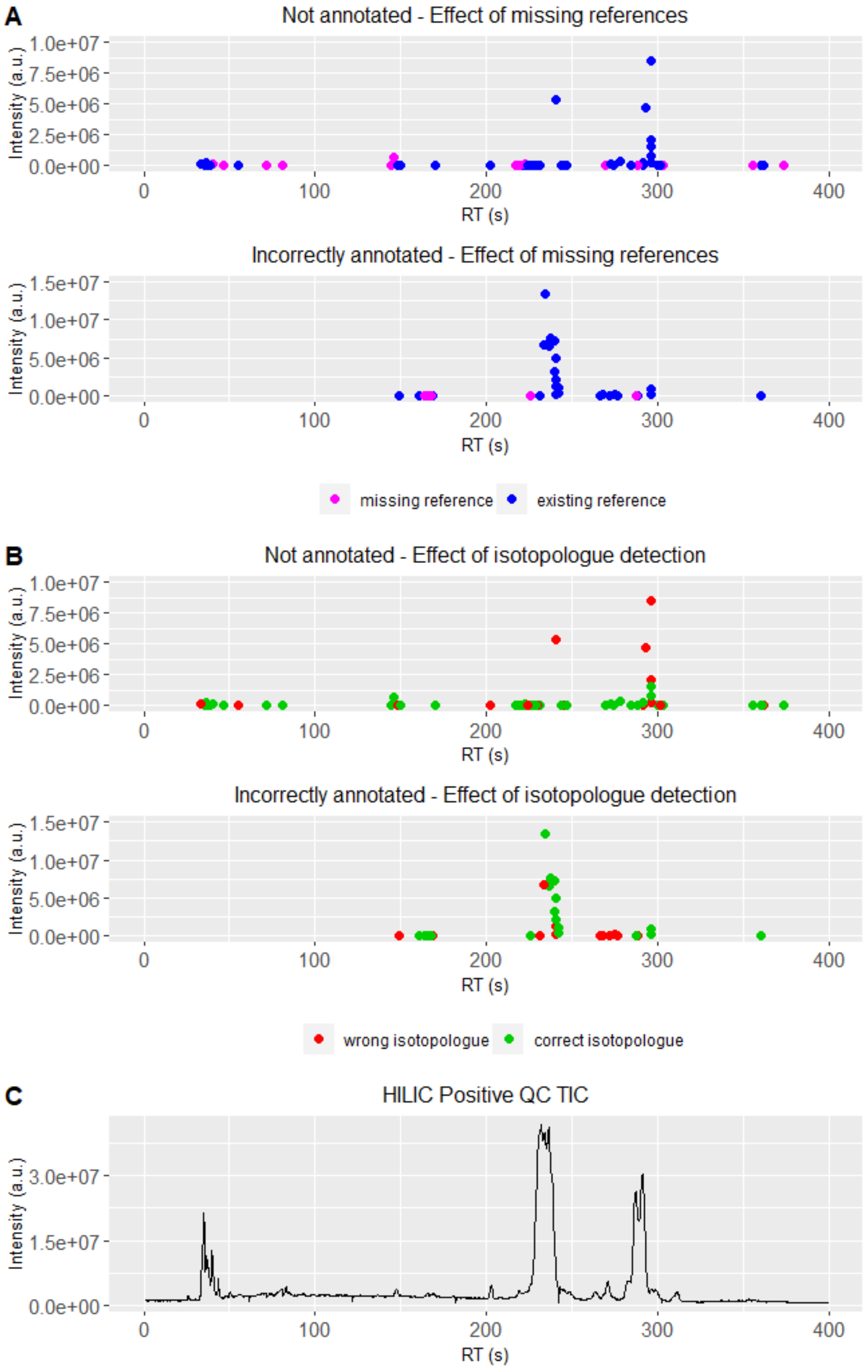

Figure S5 - Effect of missing library candidates(A) and wrongly determined isotopologue (B) in automated annotations of HILIC+ QC sample. Total ion chromatogram of the HILIC+ Serum QC sample (C) is shown to highlight regions of high ion density.

References

(1) Burke, G., Lima, J., Wong, N. D., Narula, J. The multiethnic study of atherosclerosis. *Global Heart* **2016** 11, 267-268. https://www.mesa-nhlbi.org/

(2) Olson, J. L., Bild, D. E., Kronmal, R. A., Burke, G. L. Legacy of MESA. *Global Heart* **2016**, 11, 269-274.

(3) Plumb, R.S., Johnson, K.A., Rainville, P., Smith, B.W., Wilson, I.D., Castro-Perez, J.M. and Nicholson, J.K. UPLC/MSE; a new approach for generating molecular fragment information for biomarker structure elucidation. *Rapid Commun. Mass Spectrom.* **2006**, 20: 1989-1994.

(4) Vorkas P. A., Abellona, U. M. R., Li, J. V. Tissue Multiplatform-Based Metabolomics/Metabonomics for Enhanced Metabolome Coverage. *Methods Mol. Biol.* **2018**, 1738, 239-260.
